# Impact of exercise video-guided bodyweight interval training on psychophysiological outcomes in inactive adults with obesity

**DOI:** 10.3389/fphys.2025.1527171

**Published:** 2025-04-03

**Authors:** Gabriella F. Bellissimo, Alyssa Bailly, Kelsey Bourbeau, Christine Mermier, Anthony Campitelli, Quint Berkemeier, Jonathan Specht, Jessica Smith, Jeremy Ducharme, Matthew J. Stork, Jonathan P. Little, Len Kravitz, Flávio de Castro Magalhães, Ann Gibson, Fabiano Amorim

**Affiliations:** ^1^ The College of Idaho, Caldwell, ID, United States; ^2^ University of New Mexico, Albuquerque, NM, United States; ^3^ University of Northern Iowa, Cedar Falls, IA, United States; ^4^ SUNY Oneonta, Oneonta, NY, United States; ^5^ University of British Columbia, Okanagan, Kelowna, BC, Canada

**Keywords:** bodyweight exercise, interval training, obesity, cardiorespiratory fitness, muscular strength, enjoyment, self-efficacy

## Abstract

**Purpose:**

Determine the impact of a 6-week YouTube-instructed bodyweight interval training (BW-IT) program on cardiometabolic health, muscular strength, and factors related to exercise adherence in adults with obesity.

**Methods:**

Fourteen adults (30.7 ± 10.3 yrs, BMI 35.5 ± 5.4 kg/m^2^) participated in this study. The BW-IT program progressed bi-weekly from a 1:3 to 1:1 work-to-rest ratio, using maximum effort intervals of high knees, squat jumps, scissor jacks, jumping lunges, and burpees. Pre- and post-intervention measures included peak oxygen consumption ( $\dot{V}$ O_2peak_), relative quadriceps isometric muscular strength, waist circumference (WC), body composition via bioelectrical impedance, and cardiometabolic blood markers (blood glucose, insulin, lipid panel, and C-reactive protein). Self-efficacy (task and scheduling) and physical activity enjoyment (PACES) were also assessed.

**Results:**

Relative isometric muscular strength increased by 12.5% (*p* = 0.02, dz 0.4) and absolute $\dot{V}$ O_2peak_ by 4.2% (*p* = 0.03, *dz* = 0.2). WC reduced by 2.1% (*p* < 0.001, *dz* = 0.2). Task self-efficacy was similar pre- to post-intervention (*p* = 0.53, *dz* = 0.2), while scheduling self-efficacy was reduced (*p* < 0.004, *dz* = 1.1). PACES scores were 9.6% higher week one compared to week six of BW-IT (*p* = 0.003, *dz* = 0.6). No changes occurred in body composition or cardiometabolic blood markers.

**Conclusion:**

In previously inactive adults with obesity, 18 sessions of YouTube-instructed bodyweight interval training elicited small to moderate effects on lower extremity muscular strength, cardiorespiratory fitness, and waist circumference. Future studies may benefit from longer interventions and adding a greater variety of calisthenics to determine interventions that improve physiological health and maintain or enhance factors associated with exercise adherence.

## Introduction

Obesity is defined as having a body mass index (BMI) value of ≥30 kg/m^2^ (World Health Organization, 2021). Currently, approximately 42% of adults in the United States are considered to have obesity (Centers for Disease Control and Prevention, 2022), and it is predicted to increase to 49% by 2030 (Ward et al., 2019). Obesity is associated with premature mortality and exacerbates the risk of several chronic diseases, including hypertension, type 2 diabetes, and cardiovascular disease (Jastreboff et al., 2019). Thus, prevention and treatment strategies for obesity present one of the biggest medical challenges of the 21^st^ century.

Exercise is a key intervention for mitigating cardiometabolic risks in obesity; however, some forms of exercise may be more efficacious than others. A systematic review and meta-analysis by Batrakoulis and colleagues concluded that for adults with overweight/obesity, multimodal combined training (CT) (i.e., continuous aerobic exercise plus resistance exercise) is superior to single modality training for improving cardiometabolic and physical health parameters (lipid profile, homeostatic model of insulin resistance (HOMA-IR), blood pressure, body composition, cardiorespiratory fitness (CRF), and muscular strength) (Batrakoulis et al., 2022). The second most effective mode noted was hybrid training (HT), a multi-component exercise in which the cardiovascular and musculoskeletal systems are engaged simultaneously (e.g., functional high-intensity interval training), with less time requirement than CT (Batrakoulis et al., 2022). A caveat of both CT and HT protocols is that they often require exercise equipment (e.g., agility ropes, ergometers, barbell rigs, etc.) and lack of time, access to exercise facilities/equipment, poor body image, and fears of embarrassment and stigma are common reported barriers to exercise among adults with obesity (Collins et al., 2022; Hamer et al., 2021; Korkiakangas et al., 2009). Thus, CT and HT may not be feasible or attractive for some individuals facing such barriers.

Bodyweight interval training (BW-IT) may serve as a practical and appealing option for adults with obesity to participate in cardiovascular and muscle strengthening exercise. This style of training is like HT in that it requires full-body movements using one’s body weight as resistance against gravity (Batrakoulis et al., 2021). However, unlike HT, BW-IT does not require the use of adjunct equipment, such as those mentioned above (Batrakoulis et al., 2021). Additionally, the intervals of BW-IT are often prescribed for as many repetitions as possible followed by recovery periods (Machado et al., 2019), a structure similar to well-studied styles of high-intensity interval training (HIIT) performed on cycle ergometers or treadmills (MacInnis and Gibala, 2017). Due to these characteristics, BW-IT may elicit similar cardiometabolic benefits to HIIT and HT in adults with obesity (Al-Mhanna et al., 2024; Batrakoulis et al., 2021; Batrakoulis and Fatouros, 2022). Moreover, it holds promise for promoting exercise adherence since bodyweight training, and on-demand exercise classes are currently ranked among the top global health and fitness trends (Newsome et al., 2024).

The purpose of the present study was to evaluate the effects of a 6-week, YouTube-instructed BW-IT program in physically inactive adults with obesity. The primary variables of interest included peak oxygen uptake ( $\dot{V}$ O_2peak_), isometric muscular strength, and cardiometabolic biomarkers. In addition, we aimed to understand psychological factors related to our BW-IT protocol, including enjoyment, task self-efficacy and scheduling self-efficacy.

## Methods

### Study design

Participants visited the Exercise Physiology laboratory at the University of New Mexico on two separate visits (baseline and post-intervention). Prior to each visit, participants were instructed to arrive in a hydrated and fasted state (no food or caffeine for ≥8 h) and abstain from alcohol and vigorous exercise for 24 h. During the baseline visit, written consent was obtained, and baseline data were collected (anthropometrics, body composition, CRF, maximal isometric strength, and cardiometabolic markers). In addition, participants were familiarized with the study protocol and given a wrist-based wearable technology device (Fitbit Inspire™) to measure heart rate (HR) during remote training sessions. After all fasted measurements were taken, a light snack (1–2 granola bars) was provided. Following the 6-week intervention, participants reported back to the lab for the post-intervention data collection visit comprised of the same variables collected at the baseline visit.

### Participants

The inclusion criteria were: 1) adults between 18 and 55 years; 2) classified with obesity (BMI ≥30 kg/m^2^); 3) physically inactive (<150 min of moderate to vigorous PA per week); and 4) non-smokers. Participants completed health history and physical activity readiness (PAR-Q+) (Warburton et al., 2021) questionnaires. If a participant indicated signs or symptoms of cardiovascular, metabolic, or renal disease, medical clearance was required prior to participation (Riebe et al., 2015). Participants who reported taking glucose or lipid-lowering medications were excluded from the study. To exclude individuals with unknown/potential prediabetes or Type II diabetes, a fasting (≥8hrs.) blood capillary glucose sample was collected from the finger using a lancet and analyzed by a portable glucose meter TRUE METRIX™ Blood Glucose Monitor (Trividia Health, Inc., United States). Individuals with blood glucose values within ±15% of 100 mg/dL were included due to the accuracy range reported by the manufacturer. Blood glucose levels were confirmed prior to proceeding with the study by sending blood samples to a local commercial lab (Quest Direct™, Albuquerque, NM, United States) for fasting blood glucose and hemoglobin A1c (HgbA1c).

G*Power Sample 3.1 software (G*power, Dusseldorf, Germany) was used to calculate the required sample size based on an *a priori* analysis for a paired samples t-test. The power analysis was conducted using the following parameters: test family difference between two dependent means (matched pairs), with power set to 0.80 and alpha at 0.05. A minimum sample (n = 14) was determined to achieve statistical power (1-β) of 0.80, using $\dot{V}$ O_2_ (mL/kg/min) as the primary dependent variable of interest. This was based on a previous assessment of $\dot{V}$ O_2_ (mL/kg/min) after 4-weeks of a home-based bodyweight exercise program in physically inactive adults with obesity (Scott et al., 2019). Participant demographics are summarized in Table 1.

**TABLE 1:** Participant characteristics pre- and post-BW-IT (*n* = 14).

Variable	PRE	Post	*p*	*dz*
Sex (M/F)	2/12	—	—	—
Age (years)	30.7 ± 10.3	—	—	—
Height (cm)	167.6 ± 8.6	—	—	—
Weight (kg)	99.8 ± 16.6	100.5 ± 17.1	0.21	0.04
BMI (kg/m^2^)	35.5 ± 5.4	35.8 ± 5.5	0.22	0.06
WC (cm)	99.9 ± 10.7	97.8 ± 10.2	<0.001	0.20
HC (cm)	121.9 ± 9.1	121.4 ± 9.4	0.60	0.05
BF (%)	43.9 ± 5.3	44.2 ± 4.9	0.42	0.06
SMM (kg)	31.3 ± 6.3	31.3 ± 5.0	0.76	0.01
LLM (kg)	16.8 ± 3.1	16.6 ± 3.2	0.17	0.05

Note. Data are presented as mean ± SD. M, male; F, female; BMI, body mass index; WC, waist circumference; HC, hip circumference; cm, centimeters; kg, kilograms; kg/m^2^, kilograms per meters squared; BF, body fat; %, percent; SMM, skeletal muscle mass; LLM, leg lean mass; *dz*, Cohen’s *d*.

### Baseline and post-testing measurements

#### Anthropometrics and body composition

Height was measured to the nearest centimeter (cm) using a stadiometer (Holtain Limited, Crymych, Dyfed, Great Britain), and weight to the nearest 0.1 kg (kg) using a digital weight scale (MedWeight MS-3900, Itin Scale Company, Brooklyn, NY, United States). Body mass index was calculated using body mass (kg) divided by the square of the height (meters). Waist circumference (WC) and hip circumference (HC) measurements were obtained according to WHO standard techniques (World Health Organization, 2011). Body composition [body weight (kg), skeletal muscle mass (SSM) (kg), BF%, and lean leg mass (LLM) (kg)] was assessed using a tetrapolar bioelectrical impedance (BIA) device (InBody 570, Biospace, INC., United States) according to manufacturer guidelines. This method was chosen to prioritize participant comfort, and previous research supports the use of BIA to provide accurate estimates of body composition in adults with obesity (Bosy-Westphal et al., 2008; Pietiläinen et al., 2013). Prior to measurement of body composition, hydration status was checked using urine specific gravity (USG) and participants with >1.02 were considered dehydrated (Armstrong, 2005) and rescheduled to reassess body composition.

#### $\dot{V}$ O_2_ peak and ventilatory threshold

Participants performed an individualized maximal incremental treadmill test (C966i, Precor Inc., Woodinville, WA, United States). The protocol used a fixed incline of 1% with speed increasing by 0.5 mph until the eighth minute. Starting speed was determined during warm-up by asking participants to briefly and gradually increase the treadmill speed to a speed that they believed they could maintain for 1 minute (perceived max speed, Vmax). After reaching the maximal speed with the eighth minute, the treadmill grade was increased by 1% per minute until volitional fatigue. During the test, heart rate was continuously recorded using a HR monitor (Polar V800, Polar Electro, Kempele, Finland). Gas exchange and ventilation (VE) were measured continuously using a metabolic cart (Parvo Medics True One 2,400, Sandy, UT, United States) while participants wore a nose clip and mouthpiece (Hans Rudolph Inc. Kansas City, MO, United States). The metabolic cart was calibrated according to manufacturer guidelines. All tests were completed within eight to 12 minutes (Yoon et al., 2007). Data were extracted using a 15-s breath running average and the highest data point was recorded as absolute (L/min) and relative (mL/kg/min) $\dot{V}$ O_2peak_ (Robergs et al., 2010). Ventilatory threshold (VT) was determined using the V-slope method (i.e., visual inspection of the point at which carbon dioxide ( $\dot{V}$ CO_2_) rose disproportionately to $\dot{V}$ O_2_ against time) (Beaver et al., 1986). Two experienced exercise physiologists determined VT ( $\dot{V}$ O_2_ at VT) and compiled results. A third research team member verified VT by visually inspecting values and making a final decision if VT varied more than 15s.

#### Maximal isometric muscular strength

Before testing, participants warmed up on a cycle ergometer at a self-selected pace for approximately 5 minutes. Maximal voluntary isometric knee extensor strength was measured using an isokinetic dynamometer on the participants’ dominant leg (Model 850–230, Universal Pro Single Chair Assembly, Biodex Medical Systems, Inc., Shirley, New York, United States). Each participant was seated on the dynamometer chair with the ankle firmly strapped to the distal pad of the lever arm. The measurements included three five-second maximal isometric contractions, with the knee joint angle fixed at 90°. Each maximal isometric contraction was interspersed with 10-s of rest. Participants performed a minimum of one submaximal practice set of the protocol with a 2-min rest period before testing, respectively. A complete knee extension corresponded to a joint angle of 0°. Peak torque (Nm) values were used for data analysis and expressed relative to body mass (kg).

#### Fasting blood samples

Fasting blood samples of approximately 15 mL were collected from an antecubital or dorsal hand vein. Samples were collected in serum separator tubes to allow the blood to clot by leaving it undisturbed at room temperature. Serum was obtained by centrifuging the tubes for 15 min (1,000 g, 22°C) (Allegra X-14R Centrifuge, Beckman Coulter, Brea, CA) and stored at −80°C for subsequent analysis. Serum concentrations of insulin, glucose, low-density lipoprotein (LDL), high-density lipoprotein (HDL), triglycerides (TG), total cholesterol (TC), C-reactive protein (CRP) were sent to a commercial laboratory (Quest Direct™, Albuquerque, NM, United States). Additionally, whole blood samples were collected in EDTA tubes for HgbA1c and sent to the same commercial laboratory. Insulin resistance was calculated using HOMA-IR (fasting insulin (uIU/mL) x fasting glucose (mg/dL)/405) (Gastaldelli, 2022).

#### Self-efficacy and physical activity enjoyment

Task self-efficacy and scheduling self-efficacy were assessed at the pre- and post-intervention time points using previously established recommendations (Bandura, 1997). The first 12 items were designed to determine participants’ confidence in repeating BW-IT task self-efficacy (both intervals and complete sessions). All items included the same stem, “Over the next 6 weeks, how confident are you in your physical capabilities to…” and the items were: “perform (4, 6, 8, 10, and 12, 30s BW-IT intervals on your own) and (1, 2, 3, 4, 5, 6, and 7 or more, sessions of BW-IT on your own)”. The subsequent seven items used the same stems and were designed to determine participants’ confidence in scheduling self-efficacy. Responses for task self-efficacy and scheduling self-efficacy were scored as a percentage from 0%, “not at all confident,” to 100%, “extremely confident”, in 10% increments. The averages for the 12 task self-efficacy and seven scheduling self-efficacy items were computed. Physical activity enjoyment of the BW-IT protocol was assessed using the original seven-point Likert, 18-item PACES questionnaire (Kendzierski and DeCarlo, 1991). Negatively worded items were reverse scored to calculate a total score from 7 to 126, with higher scores representing more positive enjoyment of BW-IT.

### BW-IT familiarization procedures

Participants were familiarized with the BW-IT exercises upon completion of baseline testing procedures. A certified exercise physiologist/personal trainer demonstrated the technique of each bodyweight exercise along with cues and explanations of proper movement mechanics. In addition, participants watched a pre-recorded BW-IT familiarization video (see Supplemental Material). Participants also received an electronic step-by-step guide with links to the YouTube workouts, the program’s general instructions, and the study timeline.

#### YouTube-based BW-IT program

Participants were encouraged to perform BW-IT on three nonconsecutive days per week across the 6-week intervention; however, they were informed that they could exercise on days that suited their schedule (even if on consecutive days). Each BW-IT video consisted of a 2-minute warm-up (30s of four calisthenics: jogging in place, side-stepping heel to glute kicks, ski jumps, and high knees or marching in place with high knees). The BW-IT protocol workouts consisted of two sets of five bodyweight exercises performed in the following order for as many repetitions as possible (AMRAP): high knees, squat jumps, scissor jacks, jumping lunges, and modified burpees (without push-ups). Each AMRAP interval was interspersed with active recovery intervals consisting of side-to-side stepping in place. The video displayed advanced and modified versions of each bodyweight exercise for participants to choose from based on their ability. A 2-min rest period was provided after the first five intervals. The protocol progressed every 2 weeks by increasing the duration of the work interval and decreasing the duration of the recovery interval (week 1–2: 30s work x 90s recovery, week 3–4: 40s work x 80s recovery, week 5–6: 60s work x 60s recovery). Shortly after each AMRAP interval (∼10–15s), participants were instructed to record their HR displayed on the Fitbit in an exercise journal. Each video was approximately 28 min; however, the protocol itself was 20 min (excluding the warm-up and 2-min rest between sets one and two).

Participants completed an exercise intensity survey using a link via Qualtrics™ after each BW-IT session. The survey required participants to log the date, week, and day the BW-IT workouts were completed. Next, they were asked to rank their overall level of exertion during BW-IT using the omnibus rating of perceived exertion scale (OMNI), with zero representing ‘extremely easy’ to ten ‘extremely difficult’. The OMNI scores were averaged for weeks one through six. In addition, participants were asked to enter the 10 HR values recorded in their exercise journal for their session that day. The HR values for each session were averaged for weeks one through six. Additionally, participants ranked their overall level of muscle soreness using a sliding scale from zero (no soreness) to one hundred (extreme soreness). Muscle soreness values were averaged for weeks 1–6. The exercise intensity survey was also used by the research team as a guide to determine if participants were staying on track with the program by completing their exercise sessions for the week.

#### Statistical analyses

All statistical analyses were performed using IBM SPSS (IBM Corp., Version 23.0 Armonk, NY, United States), except for Cohen’s *dz* which was calculated using G*Power (G*power, Dusseldorf, Germany). All data visualization was performed using GraphPad Prism v8.4 (GraphPad Software, Inc., United States). Student’s paired t-test was used to compare outcome variables measured pre- and post-BW-IT. Cohen’s *dz* was used to determine the magnitudes of effects from pre- to post-intervention and interpreted as trivial (<0.2), small (≥0.2 and ≤0.49), moderate (≥0.5 and ≤0.79), and large (≥0.8) (Cohen, 1992; Lakens, 2013). A repeated measures one-way analysis of variance (RM-ANOVA) was used to compare means of OMNI, HR, and muscle soreness for weeks one through six of BW-IT. A Greenhouse-Geisser correction was applied if a violation of sphericity was detected. Partial eta squared (η2) effect sizes were calculated and categorized as small (≤0.01), medium (0.06), and large (0.14) effects, respectively (Cohen, 1992; Lakens, 2013). All data are reported as mean ± standard deviation unless otherwise specified, and statistical significance was set at *p* ≤ 0.05.

## Ethical considerations

This single-group study, where all participants received the same intervention, was approved, by the University of New Mexico (UNM) Institutional Review Board (IRB) [reference # 21319] and adhered with the Declaration of Helsinki. Prior to participation, all subjects were informed of study objectives, procedures, potential risks, and benefits. Written and informed consent was obtained by all study participants.

## Results

### Adherence

Six individuals completed the program within 42 days (6 weeks) while seven completed the program late (within seven to 8 weeks). The individuals who completed the program later than expected reported difficulty with work/schedule conflicts and illness as reasons for falling behind. Thirteen participants completed the intervention with 100% compliance (18 total BW-IT sessions), while one completed it with 78% (14 total BW-IT sessions). No injuries from the BW-IT program were reported by participants.

### Anthropometrics and body composition

Anthropometric and body composition data are summarized in Table 1. A significant reduction in WC (*t* (13) *=* 12.1*, p* < 0.001*, dz* = 0.21) was observed pre- to post-BW-IT. No differences in hip circumference (HC), body weight, BMI, skeletal muscle mass, leg lean mass, or BF% were observed from pre- to post-BW-IT.

### Blood biomarkers

No differences in blood-based markers of cardiometabolic health were observed pre- to post-BW-IT (i.e., glucose, insulin, HOMA-IR, HgbA1c, total cholesterol, HDL, triglycerides, LDL, and CRP) (Table 2).

**TABLE 2:** Blood-based markers of cardiometabolic health pre- and post-BW-IT (*n* = 14).

Variable	PRE	POST	*p*	*dz*
Glucose (mg/dL)	87.1 ± 7.5	89.4 ± 8.8	0.37	0.27
Insulin (uIU/mL)	11.2 ± 7.5	12.1 ± 7.0	0.29	0.12
HOMA-IR	2.5 ± 1.7	2.8 ± 1.7	0.15	0.17
HgbA1c (%)	5.2 ± 0.4	5.1 ± 0.7	0.39	0.29
TC (mg/dL)	169.4 ± 34.9	165.7 ± 32.0	0.34	0.11
HDL (mg/dL)	48.4 ± 11.0	47.9 ± 11.9	0.78	0.04
TG (mg/dL)	99.5 ± 50.4	103.1 ± 38.7	0.61	0.08
LDL (mg/dL)	102.4 ± 30.5	98.0 ± 25.6	0.20	0.15
CRP (mg/dL)	6.0 ± 3.3	5.0 ± 3.1	0.12	0.31

Note. Data are presented as mean ± SD.; mg/dL, milligrams per deciliter; uIU/mL, mili-international units per liter; HOMA-IR, homeostatic model of insulin resistance; HgbA1c, hemoglobin A1c; TC, total cholesterol; HDL, high density lipoprotein; TG, triglycerides; LDL, low density lipoprotein; CRP, C-reactive protein.

### Cardiorespiratory fitness

**FIGURE 1:**
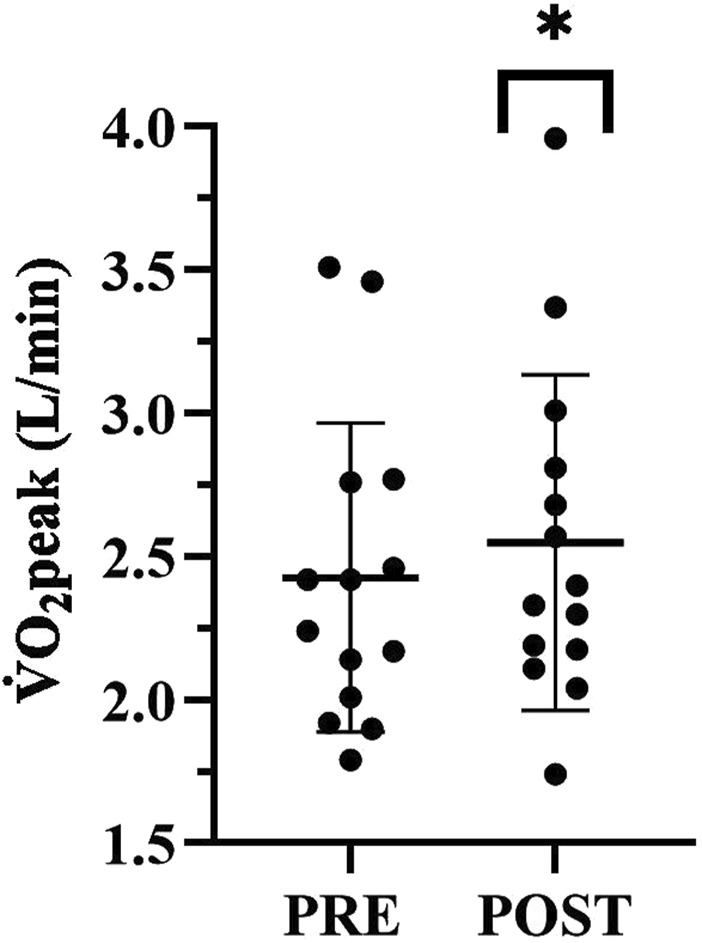
Peak oxygen consumption $\dot{V}$ O_2peak_ (L/min) pre- to post BW-IT. Data represented as mean and standard deviation, *= p < 0.05 post BW-IT.

Absolute $\dot{V}$ O_2peak_ significantly increased pre- to post-BW-IT (2.4 ± 0.5 vs. 2.5 ± 0.6 L/min, *t* (13) *=* 2.4*, p* = 0.03, *dz* = 0.2 (Figure 1). No difference in relative $\dot{V}$ O_2peak_ occurred (24.4 ± 4.1 vs. 25.5 ± 4.5 mL/kg/min, *t* (13) *=* 2.1*, p* = 0.05, *dz* 0.2). No change in VT occurred pre- to post BW-IT (1.6 ± 0.4 vs. 1.7 ± 0.4 L/min, *t* (13) *=* 1.7*, p* = 0.11, *dz* = 0.30), (15.8 ± 3.3 vs. 16.7 ± 3.3 mL/kg/min, *t* (13) *=* 1.5*, p* = 0.07, *dz* = 0.29).

### Isometric muscular strength

**FIGURE 2:**
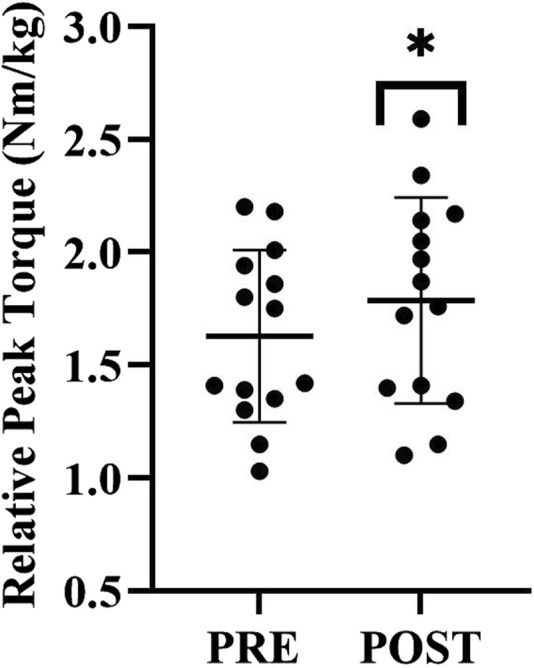
Peak torque (Nm) per body weight (kg) pre- to post BW-IT. Data represented as mean and standard deviation, *= p < 0.05 post BW-IT.

Peak torque relative to body weight significantly increased pre- to post-BW-IT (1.6 ± 0.4 vs. 1.8 ± 0.5 Nm/kg, *t* (13) *=* 2.4*, p* = 0.02, *dz* = 0.4) (Figure 2). The average coefficient of variation (CV) for isometric muscular strength was 6.0% ± 4.7% at baseline and 4.1% ± 2.2% post-testing.

### Self-efficacy and physical activity enjoyment

Task self-efficacy (i.e., confidence to perform BW-IT) did not change pre- to post-intervention (86.7% ± 12.2% vs. 83.6% ± 15.1%, *t* (13) *=* 0.7*, p* = 0.53, *dz* = 0.2). Scheduling self-efficacy (i.e., confidence to allocate time to schedule BW-IT) was significantly reduced pre- to post-intervention (94.7% ± 7.2% vs. 82.7% ± 13.5%, *t* (13) *=* 3.5*, p* = 0.004, *dz* = 1.0). Task self-efficacy and scheduling self-efficacy constructs demonstrated good internal consistency at each administration (Cronbach’s α′s ≥ 0.91). PACES scores were higher after week one (102.8 ± 16.5) compared to week 6 (92.9 ± 16.9) of BW-IT, *t* (13) *=* 3.7, *p* = 0.003, *dz* = 0.59). The PACES demonstrated good internal consistency at each administration (Cronbach’s α ≥ 0.92).

### RPE and muscle soreness

Week-by-week data for RPE, heart rate, and muscle soreness can be found in Table 3. No differences in these measurements occurred for weeks one through six: RPE (*F* (2.5, 32.8) = 1.2, *p* = 0.32, η2 = 0.08), heart rate (*F* (2.9, 37.6) = 0.7, *p* = 0.54, η2 = 0.05), muscle soreness (*F* (3.0, 38.3) = 2.7, *p* = 0.06, η2 = 0.17).

**TABLE 3:** Weekly exercise characteristics (*n* = 14).

Week	RPE (0–10)	Heart rate (bpm)	Muscle soreness (0–100)
1	7.0 ± 1.0	139.3 ± 11.9	46.6 ± 15.2
2	6.6 ± 1.6	140.5 ± 12.2	43.4 ± 18.2
3	6.9 ± 2.0	141.1 ± 12.3	44.5 ± 19.7
4	6.8 ± 2.0	141.2 ± 10.9	45.6 ± 21.5
5	7.3 ± 1.3	142.9 ± 11.7	55.6 ± 19.3
6	7.3 ± 1.4	140.7 ± 11.3	55.6 ± 20.9

Note. Data is presented as mean ± SD. RPE, rating of perceived exertion; bpm, beat per minute.

## Discussion

### Main findings

The main finding of the present study suggests that 18 sessions of bodyweight interval training (BW-IT) elicited significant, small to moderate effects on lower extremity isometric muscular strength, absolute $\dot{V}$ O_2peak_, and waist circumference (WC). Body composition did not improve (skeletal muscle mass, leg lean mass, and BF%) assessed via bioelectrical impedance (BIA). In addition, there were no changes in cardiometabolic biomarkers including fasting insulin, glucose, low-density lipoprotein, high-density lipoprotein, triglycerides, total cholesterol, C-reactive protein, hemoglobin A1c, and insulin resistance determined via the homeostatic model of insulin resistance (HOMA-IR). Our self-efficacy data suggests that participants were confident in their abilities to perform BW-IT but lacked confidence in scheduling it beyond the study duration. Moreover, the physical activity enjoyment (PACES) results suggest that these individuals may have enjoyed the shorter duration work intervals performed during week 1 (30s work x 90s recovery) as opposed to week 6 (60s work x 60s recovery) of BW-IT. It is also possible that participants were initially enthusiastic about beginning a structured exercise program in week one, but by week six, experienced a decline in motivation or engagement.

### Cardiorespiratory and muscular fitness

Previous research supports the idea that BW-IT has the potential to offer both aerobic and muscle-strengthening benefits (Archila et al., 2021; Iversen et al., 2021; Mcleod et al., 2019; Scott et al., 2019). In the present study, we used a BW-IT protocol that alternated between rhythmic, aerobic-type exercises (high knees, scissor jacks, and burpees) and muscle-strengthening type exercises, which naturally require longer time under tension (squat jumps and jumping lunges). This protocol was previously characterized in our lab as vigorous-intensity aerobic exercise in a group of healthy active, adults (Bellissimo et al., 2022). In agreement with the current findings, previous research suggests that performing high repetition (in our case, as many repetitions as possible) and low-load (in our case, using only body weight) has been reported to effectively improve CRF (Archila et al., 2021) and muscular strength (Iversen et al., 2021; Mcleod et al., 2019).

The training protocol in the present study elicited ∼4% improvement in CRF, which is slightly lower (∼3%) than the results of a previous 6-week BW-IT study in physically inactive but otherwise healthy adults (Archila et al., 2021). Furthermore, Scott and colleagues observed an 8% improvement in CRF after 4 weeks of a home-based BW-IT protocol in adults with obesity (Scott et al., 2019). Although direct comparisons between BW-IT studies are challenging due to the versatility of exercise selection, we speculate our results differ because both Archila et al. (2021) and Scott et al. (2019) employed a supervised (in-person and virtual) intervention with higher average %HR_max_ values reported during exercise. For instance, Archilia et al. reported that all sessions were performed in a supervised lab setting, and mean intensity during training was 82% ± 5% HR_max_. Further, Scott et al. (2019) supervised exercise virtually and encouraged participants to reach a target heart rate of ≥80% HR_max_ which was achieved in 99% ± 1% of exercise sessions (Scott et al., 2019). In contrast, our cohort performed all workouts independently and were instructed to perform work intervals at a self-selected relative (maximal effort) intensity. Nonetheless, even minimal improvements in CRF hold clinical relevance (Imboden et al., 2019), especially for populations like those observed in the present study who are at increased risk for cardiometabolic disease. In addition, it is important to note that 13 of our 14 participants had 100% compliance using this unsupervised protocol, suggesting that this type of intervention may be practical for use in the real world. Moreover, our task self-efficacy data suggests that participants were confident in their ability to perform BW-IT at the start and upon completion of the study.

In addition to enhanced CRF, dominant leg isometric muscular strength improved by 12.5%. These results align with those of longer duration BW-IT interventions. For example, Batrakoulis et al. (2018) report a significant progressive increase in muscular strength (1 repetition maximum leg press) at a 20-week and 40-week timepoint of BW-IT in sedentary females classified as overweight or obese (Batrakoulis et al., 2018). However, to our knowledge, there is limited evidence on the effectiveness of shorter duration BW-IT interventions on muscular strength in adults with obesity. Our findings suggest that a relatively brief intervention (18 sessions) can yield meaningful improvements in lower extremity strength. This finding is noteworthy, as lower body strength is a key predictor of functional limitations and physical disabilities with aging (Hairi et al., 2010). The importance of our findings is underscored by evidence that suggests muscular strength is an independent predictor of disease risk and all-cause mortality (Kim et al., 2018; Volaklis et al., 2015). These results highlight the potential for shorter duration BW-IT to provide meaningful lower body strength benefits.

### Body composition and cardiometabolic health

Findings from the present study indicate that apart from WC, 6-weeks of BW-IT was not sufficient to improve body composition (skeletal muscle mass, leg lean mass, and BF%) assessed via bioelectrical impedance. Therefore, the small effect observed in WC (*dz* = 0.21) should be interpreted with caution. In addition, no changes in cardiometabolic biomarkers occurred, including fasting insulin, glucose, low-density lipoprotein, high-density lipoprotein, triglycerides, total cholesterol, C-reactive protein, hemoglobin A1c, and HOMA-IR. Our results are in agreement with a meta-analytic evidence that suggests there is no effect of low-volume HIIT (≤500 MET-min/week) on body composition parameters (total body fat mass and BF%) assessed via dual-energy X-ray absorptiometry (DXA), BIA, or air displacement plethysmography (ADP) (Sultana et al., 2019). Similarly, a meta-analysis by Batacan et al. (2017) suggests that short-term HIIT interventions (<12-weeks) may be insufficient for stimulating improvements in insulin, lipid profile and C-reactive protein in adults classified with overweight/obesity (Batacan et al., 2017).

Several factors related to BW-IT make it difficult to determine its potential impact on body composition and cardiometabolic health. For example, challenges in controlling exercise intensity, individual differences in movement mechanics, and variations in exercise selection, all contribute to variability. Timmons et al. (2022) used an 8-week “Tabata-inspired” protocol (“20 s on, 10 s off”) where participants performed six different bodyweight exercises, each for 4 minutes (Timmons et al., 2022). Lean body mass increased by 2% pre- to post intervention, determined via DXA (Timmons et al., 2022). The protocol had no effect on fat mass, blood lipids, or glucose in men classified with overweight/obesity (Timmons et al., 2022). In contrast, Scott et al. (2019) found no changes in lean body mass following 8-weeks of a home-based BW-IT program, while visceral fat was reduced by 27% in adults with obesity (Scott et al., 2019). Future research is needed to determine consistencies in BW-IT protocols that elicit improvements in body composition and cardiometabolic risk factors in this population.

### Psychological outcomes

No change in task self-efficacy occurred (−3.6%, p = 0.53, dz = 0.2), while scheduling self-efficacy significantly declined (−12.7%, p = 0.004, dz = 1.0). This data suggests that participants were confident in their ability to perform BW-IT but their confidence in allocating time for BW-IT did not improve. Our results are in line with those by Dunston and Taylor (2023) who report no change in self-efficacy in previously sedentary adults after a 6-week, partially supervised, intervention of either moderate intensity continuous training (MICT) or HIIT (Dunston and Taylor, 2023). In contrast, Locke et al. (2018) observed an initial increase in self-efficacy following 2-weeks of supervised MICT or HIIT in individuals at high risk for type II diabetes (Locke et al., 2018). However, after transitioning to a 24-week unsupervised phase, self-efficacy in these participants declined, suggesting that the improvements in self-efficacy may not be maintained in free-living conditions (Locke et al., 2018). These results indicate the potential role of structured support and supervision in improving and/or sustaining self-efficacy, which may, in part, explain the lack of change observed in the present study.

In addition to the self-efficacy findings, we observed that exercise enjoyment was greater after week one (30s work x 90s recovery) compared to week 6 (60s work x 60s recovery). We speculate that the progression of the protocol may have impacted enjoyment, as it is common for adults with overweight or obesity to stop exercise when the program ramps up to higher volumes or intensities (Collins et al., 2022). Similar findings have been reported by Foster et al. (2015) who observed a progressive decline in exercise enjoyment across three 8-week exercise interventions (MICT, moderate-intensity interval training, or HIIT) in previously sedentary adults (Foster et al., 2015). Further, Foster et al. (2015) note that the lowest enjoyment was found in the most intense training protocol. It is important to note that our exercise intensity data on perceived exertion, heart rate, and muscle soreness do not suggest that the program became significantly more difficult from the first to the final sessions. Nonetheless, future BW-IT protocols using smaller incremental increases, and a greater variety of calisthenics may elicit more positive psychological outcomes. We recommend that future BW-IT training interventions incorporate small incremental increases in the duration of work intervals (e.g., 5 seconds) and a wider variety of bodyweight exercises to prevent monotony and boredom. Additionally, we suggest integrating forms of social support for interventions being performed remotely, such as positive reinforcement and virtual group sessions.

### Practical applications

Recent data show that only 28% of adults in the United States meet aerobic and muscle strengthening physical activity guidelines (≥150 min of moderate-intensity aerobic activity, ≥75 min of vigorous-intensity activity, or an equivalent combination of the two, along with ≥2 days/week of muscle-strengthening activity) (Abildso et al., 2023). We found that a relatively small volume of BW-IT (20 min/session, 60 min/week), led to improvements in both cardiorespiratory fitness and muscular strength. Notably, this improvement occurred despite participants completing the program independently. Thus, BW-IT may serve as a viable option for participating in aerobic and muscle strengthening physical activity. Our results are especially applicable to populations who report fears of embarrassment and enacted stigma as barriers to physical activity participation (Hamer et al., 2021). This protocol was instructed completely asynchronously and can be performed at locations most convenient and comfortable to the individual.

### Limitations

There are several limitations to the present study. The study was not a randomized controlled trial. The outcomes will be used to design a larger-scale study; however, the lack of randomization and a non-exercise control group increases the bias of our results. In addition, while the study was adequately powered to detect improvements in oxygen consumption, the sample size may have been insufficient to detect differences in other outcomes, such as blood biomarkers. Furthermore, although participants were classified with obesity, there was variability in the degree of obesity and the presence of cardiometabolic risk factors among individuals. We asked participants to exercise three non-consecutive days a week and aim to complete the program within 42 days (6 weeks). However, some individuals needed to postpone exercise days due to illness and schedule conflicts. Although these factors are representative of a real-world setting, they may have made an impact on the anticipated physiological and cardiometabolic responses. Participants were requested to maintain their normal diet throughout the study; however, we did not control nutritional habits. Additionally, we did not track participants’ physical activity habits throughout the study. Although we attempted to do so, compliance with regularly charging and wearing the provided FitBit devices was inconsistent, except for during exercise. Lastly, it is difficult to standardize the intensity of bodyweight exercise since biomechanics and coordination vary individually. Other BW-IT interventions may have different outcomes.

## Conclusion

Eighteen sessions of unsupervised bodyweight interval training (BW-IT) delivered via YouTube produced small to moderate effects on muscular strength, cardiorespiratory fitness, and waist circumference. Confidence allocating time to schedule BW-IT was lower after the program, while physical activity enjoyment was higher at the start. The results of this study may facilitate the use of BW-IT in previously inactive adults with obesity and highlight important variables for designing future protocols. Future BW-IT interventions that can sustain or improve factors related to exercise adherence and enhance physiological health in this population are highly warranted.

## Data Availability

The raw data supporting the conclusions of this article will be made available by the authors, without undue reservation.
