# Supplementary material for: Impact of exercise video-guided bodyweight interval training on psychophysiological outcomes in inactive adults with obesity
Source: Front Physiol. 2025 Apr 3;16:1527171. doi: 10.3389/fphys.2025.1527171 (PMC12003972; doi:10.3389/fphys.2025.1527171)
Supplement: Supplementary file 1 [file Table1.docx]

[Familiarization Video](https://youtu.be/EbeTy72VtuM)

[Exercise Videos](https://youtube.com/playlist?list=PLpqCdMYQbSNmA8zmjzuo2eVFj-IpQmFS_&si=DyG7Xw9U95bHZCy_)
